# The predictive association between radiological findings and lung cancer development in patients exposed to sulfur mustard gas: 4 decades follow up of 719 victims

**DOI:** 10.1186/s12890-022-02282-7

**Published:** 2022-12-20

**Authors:** Shahin Kavousi, Hossein Akbarialiabad, Davood Mehrabani, Alireza Mohamadian, Aria Ghahramani, Ali Shirkhoda, Reza Jalli

**Affiliations:** 1 Student Research Committee, School of Medicine, Shiraz University of Medical Sciences and Health Services, Shiraz, Iran; 2 Stem Cell Technology Research Center, Associate Professor of Pathology, Shiraz University of Medical Sciences, Shiraz, Iran; 3 Department of Radiology, School of Medicine, Tehran University of Medical Sciences, Tehran, Iran; 4 Students’ Scientific Research Center (SSRC), Tehran University of Medical Sciences, Tehran, Iran; 5 Pennsylvania State University College of Medicine, 500 University Drive, Hershey, PA 17033 USA; 6 Department of Radiology and Diagnostic Imaging, University of California, Irvine, CA 92697 USA; 7 Medical Imaging Research Center, Department of Radiology, Shiraz University of Medical Sciences, Shiraz, Iran

**Keywords:** Mustard gas, Lung neoplasms, Bronchiolitis obliterans, Pulmonary fibrosis, Bronchiectasis

## Abstract

**Background:**

Respiratory diseases are the leading cause of morbidity and mortality in the survivors exposed to Sulfur Mustard (SM). The late abnormalities can be present as chronic bronchitis, tracheobronchial stenosis, asthma, bronchiectasis, airway narrowing, lung fibrosis, and lung cancers. This study aims to investigate the association between radiological findings and lung cancer development in patients exposed to sulfur mustard gas.

**Methods:**

We entered 719 victims exposed to SM during the Iran–Iraq war into our follow-up study in a consensus manner. They were periodically followed with Chest HRCT scans from 2001 to an interval of 2014–2019. The mean year interval between exposure and the last follow-up was 38 years. For confirming the lung cancer in those with evidence of malignancy in their imaging, fine needle aspiration/biopsy and/or surgical intervention were done.

**Results:**

Among 719 patients, 57% were free from any pathologic findings in their HRCT scan. Among the subjects who had the abnormal radiologic findings, Air Trapping (AT), Lung Fibrosis (LF), Bronchiectasis (B), and the evidence of lung cancer were found in 265 (36.9%), 207 (28.8%), 151 (21.0%), and 42 (5.8%), respectively. Adenocarcinoma (38.1%) was the most common type of cancer. The right lung was involved more than the left one regarding LF, B, and cancer (*p* value < 0.05). Considering the laterality, a significant correlation was found between the side of LF and B and the tumor side. Furthermore, it was shown that the lung lobes with LF were statistically correlated to tumor-involved lobes. The relative risk of AT and B existence for tumor development was 11.73 [4.87–28.26] and 10.14 [5.12–20.090], respectively. The most predictive finding was LF which caused the risk of developing tumor 17.75 [7.35–42.86] times higher in the patient with this pathology. By each increment of the number of LF and B, the risk of developing tumors increased by 51% and 76%, respectively.

**Conclusion:**

In survivors exposed to Sulfur Mustard, those with bronchiectasis and lung fibrosis have a significantly higher risk of developing lung cancers, so a close follow-up of these victims is recommended.

*Trial registration* This study was confirmed by the institutional review board and ethics committee at Shiraz University of Medical Sciences (SUMS) with the ethical code IR.SUMS.MED.REC.1399.637.

**Supplementary Information:**

The online version contains supplementary material available at 10.1186/s12890-022-02282-7.

## Background

2,2-Dichlorodiethyl sulfide or Sulfur Mustard (SM) is an oil-soluble blistering agent which is not readily available in the environment [1]. It has a fishy odor and its color changes from clear to pale brown-yellow as a liquid and then becomes amber as a solid [2]. It is absorbed through the skin and mucus membranes and therefore compromises the barriers of eye, skin, and respiratory systems [3]. In the acute phase of injury, the corneal epithelium is the most sensitive tissue to SM [4]. Moreover, immunological, neurological, developmental, gastrointestinal, hematological, and infertility side effects have been reported as a result of SM exposure [5–9]. Long-term adverse effects include physical anomalies, other disorders in the children of victims, and a devastating social and financial burden on the families of those affected [10].

The SM is a potent alkylating agent which seems to interact with DNA, proteins, and lipids. It damages the cells in the acute phase of injury which leads to adenosine triphosphate (ATP) depletion, cell cycle arrest, apoptosis, and cell death. Moreover, it increases the calmodulin and intracellular calcium levels and decreases Glutathione [11]. The damage in chronic phase is possibly mediated by cytotoxic and oxidative effects such as DNA oxidation, nicotinamide adenine dinucleotide (NAD) depletion, and antioxidant depletion, and progressive inflammation. Passing the time, the proteinase pathways, such as caspases and matrix metalloproteinases (MMPs), are also affected [12]. While, the exact pathophysiology is under debate, it is believed that the SM generally disrupts the cell cycle via the increased oxidative stress and reduced the antioxidant capacity [13, 14]. In addition, SM is a proven carcinogen in either single or chronic exposures [15]. It is shown that SM would cause mutations in DNA of the patients, in tumor suppressor and oncogenes, such as p53 or KRAS [16, 17]. Moreover, DNA sequencing data showed that mutations on p53 were nucleotide substitutions (mostly G to A transitions) which is mediated through alkylation of DNA by highly reactive episulfonium ion [15, 18].

The respiratory problems are the most profound causes of long-lasting disability in the patients who exposed to SM. While, the exact pathophysiological mechanism of SM-mediated lung injury is still unclear, it is accepted that 80% of the inhaled SM is absorbed via the respiratory tracts [19]. The SM damages the respiratory tract in a dose-dependent manner. In a low-dose, it only affects the nasal cavity. Moreover, with more extensive doses, it may damage the lower respiratory tract including the terminal bronchioles [20]. The SM is often fatal in the acute phase (short-term) which may lead to numerous chronic phase disorders [21]. The respiratory consequences are the primary and leading source of mortality following exposure to a sufficiently high concentration of mustard [21]. The predominant respiratory symptoms following exposure include the ache and discomfort in the nostrils and sinuses with accelerated nasal secretions, hoarseness, sore throat, a burning sensation of the vocal cords, dyspnea, and intense hemorrhagic irritation and erosion of the tracheobronchial mucosa [11]. Other manifestations include bloody cough and sputum, chest pain, diminished lung sounds, and cyanosis [22]. The mucosal necrosis associated to inflammation, is expected in the most cases which can develop a membrane and subsequent blockade of airways [23].

In the acute phase, the involvement of respiratory tract in these patients can range from a nonspecific inflammation of mucosa and submucosa to a condition of closely resembling Acute Respiratory Distress Syndrome (ARDS) and even death [24]. After 6 months of chemical exposure and surviving the acute phase, the chronic phase can be present with the chronic problems such as chronic bronchitis, tracheobronchial stenosis, asthma, bronchiectasis, airway narrowing due to scarring, pulmonary fibrosis, and rarely lung cancers [18, 25]. Emad et al. [26] studied the long-term Broncho alveolar Lavage (BAL) fluid of veterans exposed to the SM that found an ongoing inflammatory process in airways which resulted in the pulmonary fibrosis.

High-Resolution Computed Tomography (HRCT) scan was used as choice method to examine the respiratory tract changes in those who exposed to SM [24, 27]. Hence, for the diagnosis of lung cancer, pathology findings are the gold standard [28].

During Iraq-Iran war (1980–1988), about 100,000 Iranian people were affected by Mustard gas [29]. Some died in the early phase, but many of the survivors are still suffering from the long-lasting impacts of toxic agents [30]. In this study, our specific purpose was to evaluate the long-term radiologic findings of lungs exposed to SM. Moreover, we tested our hypothesis to predict possible lung tumor formation due to the associated radiologic findings.

## Material and methods

All methods were performed in accordance with the relevant guidelines and regulations and informed consent was obtained from participants as approved by our institution. 1644 documented veterans were referred by Janbazan and Shohada Foundation for low dose chest CT scan followup to Namazi hospital, affiliated to Shiraz University of Medical Sciences. They were entered into our follow-up study in a consensus manner. All of them were clinically proven cases of exposure to the SM who were identified and officially recorded when they were admitted due to acute phase of exposure to SM. Our exclusion criteria were those with any history of smoking, chest trauma and radiation, primary cancer elsewhere in their body, collagen vascular disease, asthma, other hypersensitivities, and chronic lung infections such as tuberculosis and any occupational disease. Based on the above criteria, 925 individuals were excluded and therefore, 719 people were included in our study. All of them were male, and their mean age was 54 (47–73). All of the patients were followed radiologically with low dose CT scan annually or once in 3 years, Depending on the involvement of their lung especially with lung fibrosis. The radiological follow up started from 2001 to the last follow up in 2019 or to the date, they were diagnosed with cancer (interval of 2014–2019). The mean year interval between exposure and the last follow-up was 38 years.

### Imaging and pathological studies

The imaging protocol was HRCT scan (in both expiration and inhalation), 1.2 mm slice thickness, and 15 mm section interval. Bronchiolitis Obliterans (BO) is defined as multiple conditions of inflammatory pulmonary disorders, especially those affecting small airways. As a result of gas retention in the lungs, air trapping is observed when there is a discrepancy between volume or attenuation between the expiratory and inspiratory phase of respiration in chest imaging. We consider more than four air trapping as pathologic findings suggestive of bronchiolitis obliterans [31]. If there was any probability for lung malignancy, a post-contrast spiral Computed Tomography (CT) scan was done as well. To confirm the lung malignancy, fine needle aspiration/biopsy or surgical intervention were done.

### Statistical analysis

The chi-square test was applied to find the association between the radiological findings and cancer development. An independent sample *t* test was also performed to test the difference among the radiological findings. Furthermore, logistic regression was used to obtain a final model. Statistical significance was assumed if *p* value < 0.05. All reported *p* values are two-sided. All statistical analyses were performed using SPSS (version 25.0.0).

## Results

**Fig. 1:**
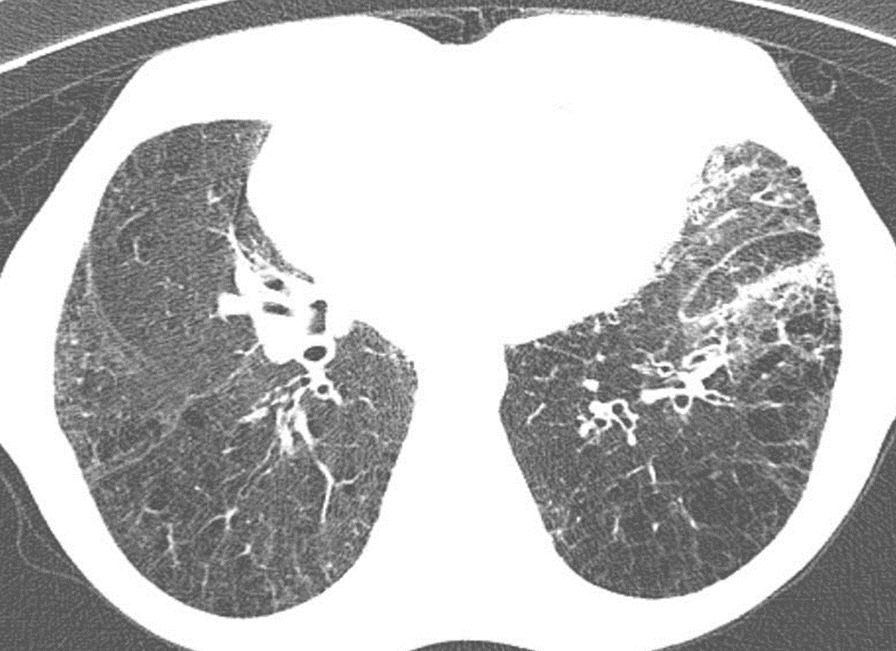
Fibrotic changes, bronchiectasis and air trapping in the lungs (more obvious on the left side)

**Fig. 2:**
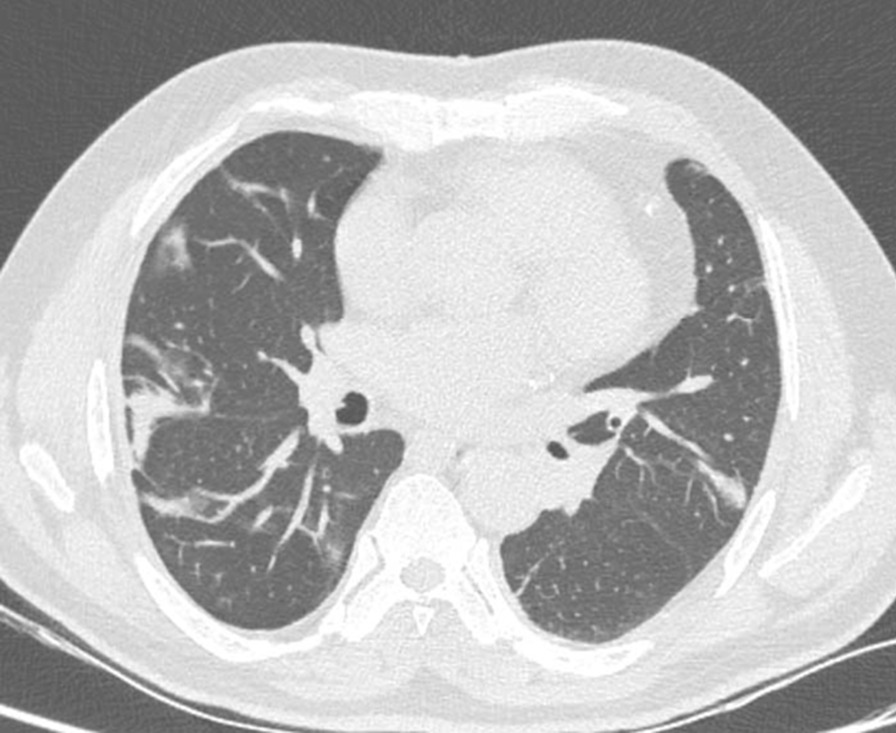
Fibrotic changes in the lungs

**Fig. 3:**
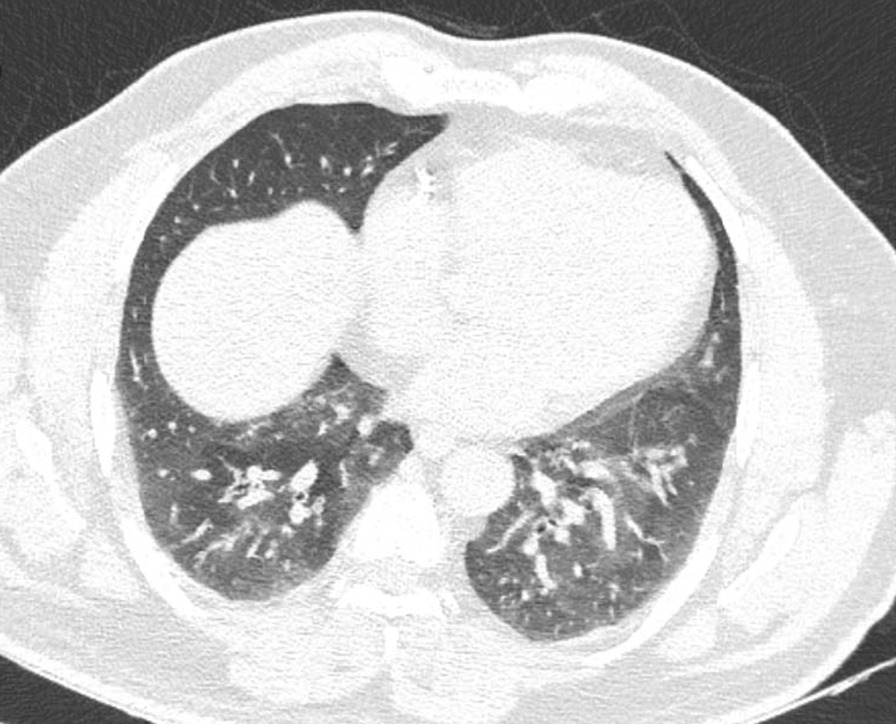
Patchy areas of air trappings in the lungs

**Fig. 4:**
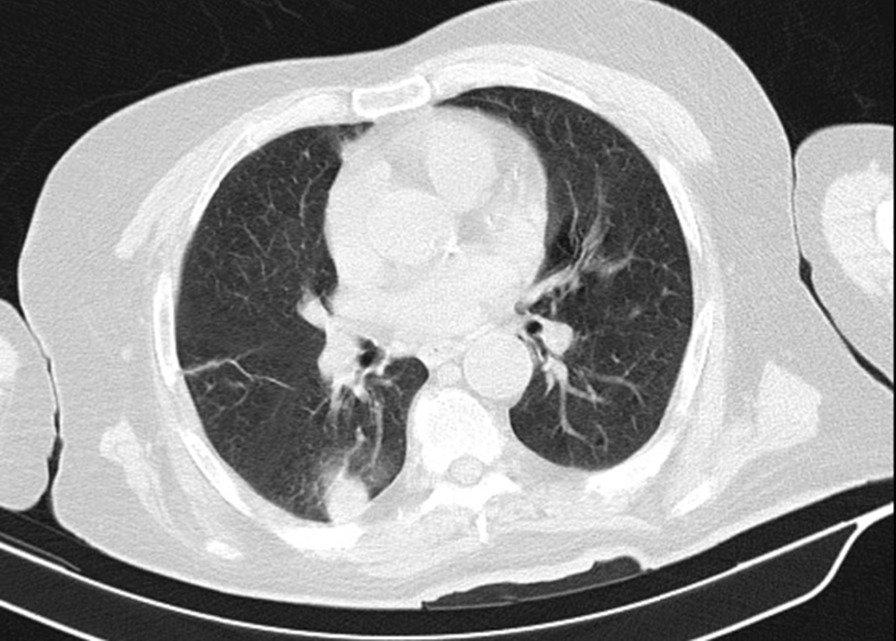
Peripherally located lesion with proved pathology of adenocarcinoma

719 patients (all male) were eligible to enter our study. Their mean age was 54 (47–73). The mean year interval between exposure and this follow-up was 38 years. Among 719 patients finally confirmed to enter the study, 151 (21%) had bronchiectasis (Fig. 1), 207 (30%) presented with lung fibrosis (Fig. 2), 265 (37%) had bronchiolitis obliterans (or pathological air trapping defined as more than four air trapping in imaging) (Fig. 3), and 42 (5.8%) showed the evidence of lung cancer (Fig. 4). It is notable to say, 57% were free from any changes in their HRCT (The raw data gathered in this research are provided in Additional file 1). As mentioned, we can consider the presence of 0–4 air trapping as physiologic in imaging; however, beyond four is pathological which considered the bronchiolitis obliterans. Concerning lung fibrosis and bronchiectasis, any sign of presence indicates the abnormality. In such a case, the maximum number of each pathology is provided as well (Table 1).

**Table 1:** Frequency of radiological findings and their percentage in 719 veterans

	Presence	Absence	Max number of pathologies
Air trapping	265 (36.9%)	454 (63.1%)	18
Lung fibrosis	207 (28.8%)	512 (71.2%)	5
Bronchiectasis	151 (21.0%)	568 (79.0%)	5
Evidence of lung cancer	42 (5.8%)	677 (94.2%)	–

The evidence of lung cancer was detected in 5.8% of participants. Its most common site was the center of right lung. After the detection of tumors, the patient underwent biopsy, and the most common type was adenocarcinoma (38.1%), followed by Squamous Cell Carcinoma (SCC) (23.8%) (Table 2).

**Table 2:** Frequency of tumors in 42 cases of lung cancer approved by surgical biopsy

Tumor type	Frequency	Percent
Atypical carcinoid	2	4.8
Adenoid cyst carcinoma	1	2.4
Adenocarcinoma	16	38.1
Large cell carcinoma	5	11.9
Mucoepidermoid Carcinoma	1	2.4
Neuroendocrine tumor	5	11.9
Typical carcinoid	2	4.8
Squamous cell carcinoma	10	23.8

One of our assumptions was that there are differences among the presence of pathologies in each lung. In all three radiological findings, the right lung was more involved than the left lung (cumulatively 390 vs. 275). Among 147 patients, pathological Air Trapping (AT) was observed in the right lung, and the number for the left lung was 118. Although the number of air-trapping views was more in the right lung, this finding was not meaningful (*p* value > 0.05). The right lung was more affected in the case of Lung Fibrosis (LF) and Bronchiectasis (B) (124 and 91 cases, respectively). Contrary to air trapping, for both lung fibrosis and bronchiectasis, there was a significant difference between the involvement of right and left lungs (*p* value < 0.05). Besides these findings, the tumor was also more observed in the right lung [28] which was also statistically significant (*p* value < 0.05) (Table 3).

**Table 3:** Frequency of each radiological findings in both lungs

	Right lung	Left lung	Expected number	*p* value	Correlation with tumor location
Air trapping	147	118	132.5	0.07	0.252
Lung fibrosis	124	83	103.5	0.004	0.004
Bronchiectasis	91	60	75.5	0.01	0.000
Tumor	28	14	21	0.03	–

These tables show that lung fibrosis, bronchiectasis, and tumor tend to develop more in the right lung. Moreover, cancer significantly develops in the lungs with radiological findings suggestive of lung fibrosis or bronchiectasis

Following the preliminary result, we performed a parametric chi-square test to determine whether there is a relationship between the location of tumor and other radiological findings. Moreover, there was no statistically significant correlation between the involvement of right and left lungs and the tumor site for AT. However, there was a significant correlation between the location of lung fibrosis and bronchiectasis with the location of tumor (Table 3).

Other assumption of study was that there is a difference between the afflictions of each lobe by nitrogen mustard. As indicated below, there was a difference among the lobes involved with each radiological findings. The lobes which were the most involved in each air trapping, lung fibrosis, and bronchiectasis were LLL (24.5%), RML (27.1%), and RML (36.4%), respectively. The difference between the number of cases showing the involvement of each lobe with air trapping was not significant, but both lung fibrosis and bronchiectasis showed significant differences. However, the location of tumor was not significantly different among multiple lobes (Table 4).

**Table 4:** Distribution of radiological findings in each lobe and tumor locations

	AT cases (%)	LF cases (%)	B cases (%)	T cases (%)
RUL	56 (21.1)	43 (20.8)	21 (13.9)	5 (11.9)
RML	37 (14)	56 (27.1)	55 (36.4)	4 (9.5)
RLL	54 (20.4)	25 (12.1)	15 (9.9)	8 (19)
LUL	53 (20.0)	45 (21.7)	36 (23.8)	6 (14.3)
LLL	65 (24.5)	38 (18.4)	24 (15.9)	2 (4.8)
R.C	–	–	–	11 (26.2)
L.C	–	–	–	6 (14.3)
*p* value	0.10	0.015	0.000	0.21
Correlation with tumor site	1.000	0.006	0.090	

This table shows bronchiectasis and lung fibrosis are more frequently found in RML also demonstrate the lobe involved with LF can be the location of the future tumor

*AT* air trapping, *B* bronchiectasis, *LF* lung fibrosis, *T* tumor, *R.C* right central, *RLL* right lower lobe, *RML* right middle lobe, *RUL* right upper lobe, *LUL* left upper lobe, *L.C* left central, *LLL* left lower lobe

Then, we performed a chi-square test to determine a correlation among the involved lobes with the tumor and radiological findings. To perform the test, we have to omit the cases whose tumor was detected for them in R.C and L.C.; it was shown that the only lung fibrosis-involved lobes were statistically correlated to tumor-involved lobes (*p* value < 0.05) (Table 4).

Then, we performed a *t* test to determine if there is a difference between cancerous and non-cancerous patients regarding the number of pathologies. The mean number of AT detected in cancer patients was around nine, while it was around six in non-cancerous patients. There was a significant difference between the mean number of AT [2.76; CI 1.61–3.91], LF [1.46: CI 1.1–1.82], and B [1.09; CI 0.85–1.34] in the cancerous participants and tumor-free ones. Hence, the higher number of these views are detected, the greater risk is to develop cancer (Table 5).

**Table 5:** Mean number of radiological finding in cancerous patient and cancer-free ones

	Tumor	N	Mean	SD	Std. error mean
Air trapping number	Not exist	677	6.16	3.594	.138
	Exist	42	8.93	3.598	.555
Lung fibrosis number	Not exist	677	.58	1.141	.044
	Exist	42	2.05	1.431	.221
Bronchiectasis number	Not exist	677	.31	.744	.029
	Exist	42	1.40	1.231	.190

Then, we handled a chi-square test between the mere existence of all three described pathologies (regardless of how many) and developing tumors. The presence of each one had a significant relationship with the existence of a tumor. The relative risk of AT existence was 11.73 (4.87–28.26) and 10.14 [5.12–20.090] for bronchiectasis existence. The most predictive finding was LF, causing the risk of developing tumor 17.75 [7.35–42.86] times higher in the patient with this pathology.

Finally, we performed logistic regression to determine if the number of these findings can predict the development of tumor. The effect of the number of AT was not significant, although both LF and B significantly impact developing tumors. This model found that with each increment of the number of LF, the risk of developing tumors increased by 51%, and with each increment of the number of B, the risk increased by 76% (Table 6).

**Table 6:** Logistic regression model, which shows (sig < 0.05) the number of LF and B lesions, can increase the risk of developing tumors by 51% and 75%, respectively (Exp(B))

	B	S.E	Wald	*df*	Sig	Exp(B)	95% C.I. for EXP(B)	
							Lower	Upper
AT	.013	.051	.064	1	.801	1.013	.916	1.120
LF	.412	.142	8.406	1	.004	1.510	1.143	1.995
B	.565	.172	10.739	1	.001	1.759	1.255	2.467
Constant	− 3.790	.380	99.406	1	.000	.023		

## Discussion

Respiratory problems are the most profound causes of long-lasting disability in the patients who exposed to SM. This study included 719 male victims who exposed to SM, following 4 decades of exposure, with a mean age of 54 (47–73). More than half of study cases (57%) were free from any pathologic findings in their HRCT. Among the subjects who had abnormal radiologic findings, AT, LF, B, and the evidence of lung cancer were found in 265 (36.9%), 207 (28.8%), 151 (21.0%), and 42 (5.8%), respectively. The most common type of cancer was adenocarcinoma (38.1%), followed by SCC (23.8%). There was a significant difference between the involvement of right and left lung for LF, B, and cancer as they were more seen in the right lung (*p* value < 0.05). Regarding the laterality, a significant correlation was shown between the side of LF and B and the tumor side. Moreover, it was revealed that the lung lobes with LF were statistically correlated to tumor-involved lobes (*p* value < 0.05).

Despite the longer follow-up time in our study (38 years after exposure), radiographic abnormalities were observed in less than half of individuals who were exposed to SM which was considerably less than the study results of Ghanei et al. and Ratki et al., in which nearly two-thirds of their patients had HRCT abnormalities and their data were gathered 15 and 20 years after exposure, respectively [31, 32]. The present study cases were considerably more than those two studies' cases, and that disparity regarding radiographic abnormalities could be related to this difference; or, it could be simply because patients with more abnormalities had higher mortality and most of them did not reach the fourth decade after exposure. AT was found as the most frequent radiologic finding in the present study. This finding was consistent to Ghanei et al. and Emad et al.'s studies [31, 33]. Furthermore, Darchini et al. [34] showed AT was the most common abnormality in HRCTs, and 50% of their cases have some degrees of AT. AT is crucial because it shows the obstruction of small bronchioles which is probably a radiographic sign of bronchiolitis obliterans. In brief, it seems that AT is the most sensitive findings for those exposed to SM beyond 15 years after exposure [35]. The right lung is involved more than the left lung, except for AT which was not significant. While, RML was the most involved lobe in the present study; in most studies, the lower lobes of lungs were most frequently affected [32, 36, 37].

Furthermore, Darchini et al. [34] indicated Bronchiectasis (25%) and pulmonary fibrosis (25%) were other common HRCT findings. The present study results were in agreement with this study regarding these pathologies. In another study by jalli et al., the prevalence of B was 19.3%, which is compatible to the present study [37].

The mustard gas studies on the other species rather than humans revealed that pulmonary fibrosis is the primary morbidity following exposure to mustard gas [38, 39]. The studies in humans were opposite to these findings [35]. Emad and Rezaeian [33] found around one-eighth (12%) of 197 veterans progressed with lung fibrosis 10 years after exposure. Balali et al. [40], when studied the victims 25 years after exposure, found that only 7.7% had pulmonary fibrosis. These findings are inconsistent to our findings as we found that the frequency of lung fibrosis was high as one-third (28.8%) of the exposed population. It possibly occurred because we studied these survivors around 4 decades after the exposure, and LF development may be raised in the long-term.

Large nodules or mass-like lesions were not detected in Darchini et al.’s study which can be due to their small sample size [34]. It is found that about 6 percent of victims had some evidence of lung malignancy detected through the HRCT. It is speculated that the overproduction of reactive oxygen species and oxidative stress in injured regions from the mustard lungs are responsible for tumorigenesis and increased lung cancer risk [41]. Considering the count of pathologies, AT [2.76; CI 1.61–3.91] and LF [1.46: CI 1.1–1.82] were more detected in the lung of cancerous participants compared to tumor-free ones. In patients with abnormal radiological findings compared to those without them, the relative risk of AT and B existence for tumor development was 11.73 [4.87–28.26] and 10.14 [5.12–20.090], respectively. The most predictive finding was LF, causing the risk of tumor development 17.75 [7.35–42.86] times higher in the patient with this pathology than those patients who doesn’t show it. Finally, in patients exposed to SM, by each increment of the number of LF and B, the risk of developing tumors increased by 51% and 76%, respectively. In fact, the lung fibrosis location could be useful to predict the location of future tumor.

Previous studies confirmed that those who had exposure to mustard gas had a higher risk of lung malignancy. By evaluating the survivors of the first world war, it was shown that the risk of cancers is elevated in those who exposed to nitrogen mustard [42]. Nishimoto et al. studied 1632 workers of SM factory from 1929 to 1945 which followed to 1980. They found that the risk of cancer development is nearly fivefold among people who were exposed to SM [43]. Other study in 20 Iranian veterans was suggestive of the carcinogenic effect of mustard [18]. A large cohort composing of 15,000 with/without a history of exposure with 25-year follow up suggested that the rate of the different types of cancers is significantly higher as cancer risk ratio of 2.2 (1.41–2.88, 95% CI) [44] In that study, the tumor rate was 5/7570 and 2/7595 in exposed and control group, respectively. The writers of that study believed that more studies are required to address the pulmonary cancers in mustard gas survivors.

Several limitations should be considered to interpret the results. There was no control group in this study. Moreover, the amount and level of exposure and intensity of the symptoms and radiological findings in the acute phase of injury were not available. Besides, the laterality of results may be related to different sizes of right and left lungs. For prospective studies, we suggest the volume of lungs should be calculated for each case; then, the number of pathologies should be adjusted considering the volume of lungs of inflicted population to interpret the results better.

## Conclusion

In conclusion, this study contributes to our knowledge considering the long-term devastating effects of mustard due to the bronchiolitis obliterans, bronchiectasis, and especially lung fibrosis and lung tumors. Those with bronchiectasis and lung fibrosis have significantly higher chances of developing lung cancers, so a close follow-up of these victims with low dose chest CT scans is warranted.

## Supplementary Information

**Additional file 1**. The raw data gathered in this research.

## Data Availability

All data generated or analyzed during this study are included in this published article [and its supplementary information files (Additional file 1)].

## Abbreviations

ADE: Adenocarcinoma
ARDS: Acute Respiratory Distress Syndrome
AT: Air trapping
ATP: Adenosine triphosphate
B: Bronchiectasis
BAL: Bronchoalveolar lavage
BO: Bronchiolitis obliterans
HRCT: High-Resolution Computed Tomography
L.C: Left central
LF: Lung fibrosis
LLL: Left lower lobe
LUL: Left upper lobe
MMPs: Matrix metalloproteinases
NAD: Nicotinamide adenine dinucleotide
R.C: Right central
RLL: Right lower lobe
RML: Right middle lobe
RUL: Right upper lobe
SCC: Squamous cell carcinoma
SM: Sulfur mustard
T: Tumor
